# MoSnf5 Regulates Fungal Virulence, Growth, and Conidiation in *Magnaporthe oryzae*

**DOI:** 10.3390/jof9010018

**Published:** 2022-12-22

**Authors:** Xiao-Wen Xu, Rui Zhao, Xiao-Zhou Xu, Liu Tang, Wei Shi, Deng Chen, Jun-Bo Peng, Vijai Bhadauria, Wen-Sheng Zhao, Jun Yang, You-Liang Peng

**Affiliations:** 1 MARA Key Laboratory of Pest Monitoring and Green Management, College of Plant Protection, China Agricultural University, Beijing 100193, China; 2 Hubei Academy of Forestry, Wuhan 430074, China

**Keywords:** rice blast fungus, pathogenesis, SWI/SNF complex, autophagy, galactose metabolism

## Abstract

Snf5 (sucrose nonfermenting) is a core component of the SWI/SNF complexes and regulates diverse cellular processes in model eukaryotes. In plant pathogenic fungi, its biological function and underlying mechanisms remain unexplored. In this study, we investigated the biological roles of MoSnf5 in plant infection and fungal development in the rice blast pathogen *Magnaporthe oryzae.* The gene deletion mutants of *MoSNF5* exhibited slower vegetative hyphal growth, severe defects in conidiogenesis, and impaired virulence and galactose utilization capacities. Domain dissection assays showed that the Snf5 domain and the N- and C-termini of MoSnf5 were all required for its full functions. Co-immunoprecipitation and yeast two-hybrid assays showed that MoSnf5 physically interacts with four proteins, including a transcription initiation factor MoTaf14. Interestingly, the *∆MoTaf14* mutants showed similar phenotypes as the *∆Mosnf5* mutants on fungal virulence and development. Moreover, assays on GFP-MoAtg8 expression and localization showed that both the *∆Mosnf5* and *∆MoTaf14* mutants were defective in autophagy. Taken together, MoSnf5 regulates fungal virulence, growth, and conidiation, possibly through regulating galactose utilization and autophagy in *M. oryzae*.

## 1. Introduction

The SWI/SNF (switching defective/sucrose non-fermenting) complex is a conserved ATP-dependent chromatin remodeling complex, which plays essential roles in coordinating chromatin architecture and gene expression among eukaryotes [1]. The SWI/SNF complex usually contains over 13 subunits, in which Snf5 as the core one is essential to the execution of nucleosome remodeling [1,2,3,4,5]. Snf5 is localized in the nucleus and can be re-located into the cytosol under hypoxic conditions [6]. Snf5 can interact with SWI/SNF complex subunit Swi2/Snf2 or Brm (Brahma) [7,8], transcriptional activators c-Myc and Ebna2 (Epstein–Barr nuclear antigen 2) [9,10], acidic activation factors Gcn4 and Hap4 [11], and cell cycle regulator Bex2 (brain expressed X-linked protein 2) [12]. Moreover, Snf5 can serve as a pH sensor for the transcriptional response to carbon starvation [13].

Snf5 orthologues have been well functionally characterized in model eukaryotes. In *Saccharomyces cerevisiae*, the *snf5* mutants show reduced growth on glucose and sucrose, disability of growing on galactose or glycerol, and hypersensitivity to lithium ions, and are defective in the activated transcription of Sc*SUC2* [14,15]. In *Schizosaccharomyces pombe*, ScSnf5 participates in regulating the G2/M transition [16]. HsSnf5 plays critical roles in diverse cellular processes, including viability, development, differentiation, and apoptosis [17,18], and functions as a suppressor of malignant rhabdoid tumors and hepatocellular carcinoma in humans [19,20]. In *Drosophila*, Snf5 is essential to RNA polymerase elongation, pre-mRNA splicing regulation, and chromatin accessibility of ecdysone hormone-regulated genes [21]. In *Arabidopsis*, the Snf5 gene *BSH* could partially complement defects of yeast *snf5* mutation and regulates seed germination via the gibberellin pathway dependent on histone mono-ubiquitination 1 [22,23]. In *Candida albicans*, CaSnf5 was required for silicone adherence, biofilm formation, cell aggregation during yeast form growth, hyphal morphogenesis, and responses to cell wall integrity inhibitors [24]. CaSnf5 also serves as a major transcriptional regulator linking oxygen status to metabolic flexibility [25]. NcSnf5 was required for cell-to-cell fusion in *Neurospora crassa* [26]. CcSnf5 is indispensable for fruiting initiation and dikaryon development in *Coprinopsis cinerea* [27,28]. However, the biological roles of Snf5 orthologues in plant fungal pathogens and the underlying mechanisms remain unexplored.

The hemibiotrophic ascomycetous fungus *Magnaporthe oryzae* is the causal agent of rice blast, which is one of the most devastating diseases of rice across the world [29]. Besides its economic importance, *M. oryzae* also serves as the model plant pathogenic fungus with the most extensive studies [30]. The fungal infection cycle initiates with the conidium attached to the surface of the host plant. Next, the conidium germinates and forms a specialized infection structure at the tip, the melanized appressorium, in which a high concentration of glycerol and huge turgor pressure are generated for penetration [31,32]. After successfully penetrating into host cells, the thin primary infection hyphae differentiate as bulbous secondary invasive hyphae, accompanied by absorbing nitrogen from host cells [33]. The fungus then switches from the biotrophic stage to the necrotrophic stage, kills host cells, and generates disease lesions, on which thousands of the fungal conidia are produced for the next cycle of plant infection [34]. In this study, we investigated the biological functions of an Snf5 orthologous gene, *MoSNF5*, on fungal growth, conidiation, infection-related morphogenesis, and virulence in *M. oryzae*. We dissected the biological roles of the Snf5 domain and the N- and C-termini of MoSnf5 and also studied the roles of *MoSNF5* on galactose utilization and autophagy. Moreover, we identified one of the MoSnf5-interacting proteins and characterized its biological functions.

## 2. Materials and Methods

### 2.1. Fungal Strains, Growth Conditions, and Transformation

The wild-type strain P131, the deletion mutants of *MoSNF5* and *MoTAF14*, and their various transformants generated in this study (Table S1) were cultured at 26 °C on oatmeal tomato agar (OTA) plates [35,36]. Two-day-old mycelia shaken in liquid complete medium (CM) at 160 rpm were collected, and then used for isolating nucleic acids, proteins, or protoplasts. The isolated protoplasts were transformed using a PEG/CaCl_2_ method [37]. Media supplemented with 250 μg/mL hygromycin B (Roche, Basel, Switzerland) or 400 μg/mL neomycin (Ameresco, Framingham, MA, USA) were used for selecting hygromycin-resistant or neomycin-resistant transformants, respectively. To assess the mycelial growth and colony characteristics, fungal blocks were cultivated on OTA plates at 26 °C for 120 h. To observe vegetative mycelial growth on different carbon source media, 0.5 M of each carbon source, such as glucose, sucrose, galactose, NaAc, glycerol, and ethanol, was substituted for glucose in minimal medium as a composition described by Talbot et al. (1993). For testing cell wall integrity, mycelial plugs were cultured on solid complete medium (CM) and added with 200 μg/mL Congo red (CR, Sigma, St. Louis, MO, USA), 200 μg/mL Calcofluor white (CFW, Sigma), or 0.05% sodium dodecyl sulphate (SDS). Conidiation and plant infection assays on barley and rice seedlings were performed as previously described [35,38]. Conidiation was assayed with OTA cultures as previously described [39]. Conidia harvested from conidiation plates were used for the virulence test and infection process observation [36].

### 2.2. Nucleic Acid Manipulation

A CTAB protocol was used to extract fungal genomic DNA from vegetative hyphae [40]. Standard protocols of molecular biology were followed for plasmid extraction, DNA gel blot analyses, and DNA enzymatic digestion [41]. Probes were labeled with the random primer labeling kit (TaKaRa, Kusatsu, Japan). Recombinant DNA was sequenced with an ABI 3750 sequencer (SunBio Company, Beijing, China). Homology searches on DNA or protein sequences were performed by using BLAST. Identification of domains was performed by SMART. Primers used in this study are listed in Table S2.

### 2.3. RT-Quantitative PCR

To measure the relative expression levels of *MoSNF5* at different developmental stages, whole RNAs were extracted with an RNA extraction kit (Invitrogen, Waltham, MA, USA) from different tissues, including mycelia cultured in liquid CM, conidia produced on OTA plates, appressoria formed on hydrophobic film surfaces, and infected-barley leaves inoculated with conidial suspensions. The crude RNA was pre-treated with gDNA wiper (Vazyme, Nanjing, China) and was then reversely transcribed with HiScriptR II Reverse Transcriptase (Vazyme). Quantitative RT-PCR (RT-qPCR) was performed on an ABI 7500 real-time PCR system (Applied Biosystems, Waltham, MA, USA) according to the manufacturer’s instructions. The stable-expression actin gene (MGG_03982) was used as an internal control. For comparing the relative abundance of target transcripts, the mycelial sample was used as a calibrator and the average threshold cycle (Ct) was normalized to the actin gene as previously described [42]. When comparing the relative abundances of target genes between the wild-type strain and mutants, the wild-type sample was used as a calibrator. The experiments were repeated three times, with three replicates each time.

### 2.4. Gene Deletion and Complementation of MoSNF5 and MoTAF14

For the deletion of *MoSNF5*, a gene replacement construct pKNH-SNF5 was generated, and 1.5 kb upstream and 1.5 kb downstream flanking sequences were amplified from the P131 genomic DNA with the primer sets 6315LF/6315LR and 6315RF/6315RR. These two fragments were then ligated into pKNH with a hygromycin phosphotransferase (*HPH*) gene [43] as pKNH-SNF5, which was then used to transform the P131 protoplasts. Similarly, the 1.5 kb upstream and 1.5 kb downstream flanking sequences were amplified from the wild-type genomic DNA with the primer pairs 5204LF/5204LR and 5204RF/5204RR, and ligated into pKNH as pKNH-TAF14, which was also then transformed into the P131 protoplasts. For gene complementation, a genomic fragment of the *MoSNF5* gene with a 1.5 kb native promoter and a 0.5 kb terminator was amplified with primer pair 6315CF/6315CR and cloned into pKN [36]. After linearizing with *Dra*I, the resulting pCSNF5 was introduced into the deletion mutants of *MoSNF5*.

### 2.5. Subcellular Localization of MoSnf5

For subcellular localization analysis of MoSnf5, the coding region of *MoSNF5* fused with GFP was cloned into pKNRP27 [36], and the resulting construct was linearized with *Sca*I and transformed into the deletion mutants of *MoSNF5*. The resulting neomycin-resistant transformants were obtained and used for GFP observation.

### 2.6. Pathogenicity Test

Rice (*Oryza sativa* cv. LTH) seedlings at the fourth leaf stage and 8-day-old barley seedlings (*Hordeum vulgare* cv. E9) were sprayed with a conidial suspension of 5 × 10^4^ conidia/mL in 0.025% Tween 20 and incubated as described previously [36]. Lesion formation was examined at 7 d after inoculation (dai) for rice leaf and 5 dai for barley leaf [42].

### 2.7. Microscopy Assays

To stain the septa, mycelia or conidia were incubated in 10 μg/mL Calcofluor white (Sigma) solution for 5 min at RT followed by 3 thorough washes with sterile water. Samples were visualized and photographed as previously described [42]. To observe the fungal infection process, conidial suspension with a concentration of 2 × 10^5^ conidia/mL was dropped onto the lower epidermis of barley leaves and then incubated in a moist, dark chamber at 28 °C. Microscopy observations were performed at 12, 24, 36, and 48 hpi [44]. For autophagy assays, strains expressing GFP-MoAtg8 were cultured in liquid complete medium for 48 h, and then shifted to nitrogen starvation cultured for 4 h to induce autophagy. Mycelia were harvested from liquid complete medium and nitrogen starvation medium. 7-amino-4-chloromethylcoumarin (CMAC, Invitrogen) was used to stain vacuolar lumens as previously described [45]. Epifluorescence microscopic examinations were performed by using a Nikon Ni90 fluorescence microscope (Nikon, Tokyo, Japan).

### 2.8. Yeast Two-Hybrid Assay

A yeast two-hybrid approach was used to determine the protein–protein interactions. The bait construct was generated by cloning the full-length or partial cDNAs of *MoSNF5* into pGBKT7. The cDNAs of tested genes were cloned into pGADT7 as prey constructs. The resulting vectors were confirmed by sequencing analysis and transformed in pairs into yeast strain AH109 as described in the manufacturer’s handbook (Clontech, Mountain View, CA, USA). The resulting transformants were selected on SD-Trp-Leu and SD-Trp-Leu-His plus X-gal plates. Yeast strains harboring the pGBKT7-53/pGADT7-T and pGBKT7-Lam/pGADT7-T were used as positive and negative controls, respectively.

### 2.9. Western Blot

Mycelia (0.5 g) were ground to a fine powder in liquid nitrogen for total protein extractions and then re-suspended in 1.0 mL of extraction buffer (50 mM Tris–HCl pH 8.0, 150 mM NaCl, 0.5% TritonX-100) and 10 μL of protease inhibitor cocktail (Sigma). Lysates were cleared by centrifugation at 12,000 rpm for 20 min at 4 °C. Protein samples were then fractionated by SDS-PAGE, transferred onto a PVDF membrane (Millipore, Burlington, MA, USA), and immunoblotted with an anti-FLAG, anti-HA, or anti-GFP antibody (Abcam) [43]. For the GFP-MoAtg8 proteolysis assay, the pGFP-MoATG8 vector was transformed into the strain P131, and the deletion mutants of *MoSNF5* and *MoTAF14*, and the transformants expressing GFP-MoAtg8 were selected to detect the GFP-MoAtg8 proteolysis with an anti-GFP antibody [46]. For Western blot analysis, the total protein content was quantified using a Bradford Protein Assay Kit (Biyuntian, Shanghai, China).

### 2.10. Pull-Down and Co-Immunoprecipitation Assay

To identify MoSnf5-interacting proteins, the construct MoSnf5*-*3×FLAG was transformed into protoplasts of the deletion mutants of *MoSNF5*, and total proteins of the resulting transformants were isolated and incubated with anti-FLAG M2 beads (Sigma) as previously described [43]. Proteins bound to M2 resins were confirmed by Western blot with an anti-FLAG antibody. The elution proteins were analyzed by LC-MS/MS, and the resulting peptides were used to search against the *Magnaporthe oryzae* protein database. To confirm the interaction between MoTaf14 and MoSnf5 in vivo, total proteins were isolated from transformants co-expressing MoTaf14-GFP and MoSnf5*-*3×FLAG, and were incubated with GFP beads (Chromotek, Munich, Germany). Proteins bound to resins were eluted with washing buffer twice and collected in a fresh test tube. Western blots of total and eluted proteins with different antibodies corresponding to tags were performed to evaluate the interaction between the two tested proteins [38]. The same procedure was used for detecting the interaction between MoSnf5*-*3×FLAG and MoPcna1-GFP using GFP beads, and MoSnf5*-*3×FLAG and MoSsr3-3HA or MoSnf21-3HA using FLAG M2 beads.

### 2.11. Statistical Analysis

Comparisons between the two groups were evaluated using a *t*-test (parametric) for the experimental data. Multiplicity adjusted *p*-values are reported as * *p* < 0.05 and ** *p* < 0.01.

## 3. Results

### 3.1. MoSNF5 Is Important for Vegetative Hyphal Growth, Conidiation, and Cell Wall Integrity

**Figure 1:**
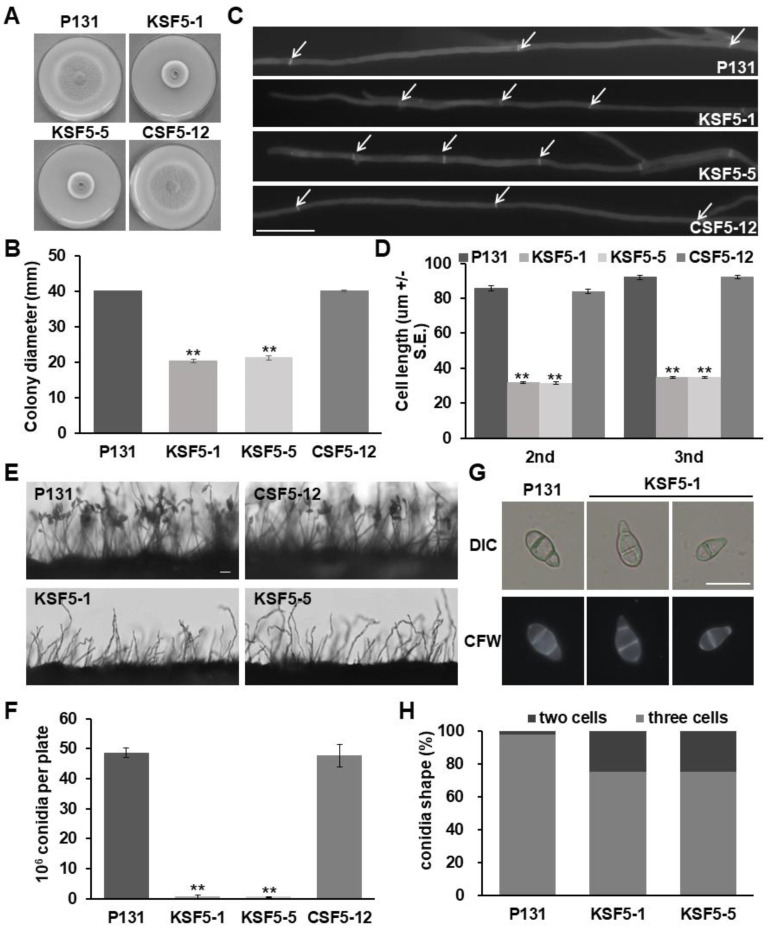
*MoSNF5* is required for vegetative hyphal growth and conidiation. (**A**) Colonies of the wild-type strain P131, the Δ*Mosnf5* mutants KSF5−1 and KSF5−5, and its one complementation strain CSF5−12 cultured on an OTA plate at 5 dpi. (**B**) Statistics on colony diameters of strains P131, KSF5−1, KSF5−5, and CSF5−12 at 5 dpi. The mean and standard deviations were calculated based on three independent experiments by measuring three plates in each replicate. Asterisk marks a significant difference between the mutants and the P131 strain by using *t*-test (*p* < 0.05). (**C**) Vegetative hyphae of strains P131, KSF5−1, KSF5−5, and CSF5−12 stained with Calcofluor white. White arrows point to the septa. Bar, 20 μm. (**D**) The average length of the secondary- and third-apical cells of vegetative hyphae of strains P131, KSF5−1, KSF5−5, and CSF5−12. The means and standard deviations were calculated by measuring 30 mycelia. Asterisk marks a significant difference between the mutants and the P131 strain by using *t*-test (*p* < 0.01). (**E**) Vertical view of conidiation structures at 24 h after induction of conidiation. Bar, 20 μm. (**F**) Capacity on conidiation of strains P131, KSF5−1, KSF5−5, and CSF5−12. The mean and standard deviations were calculated based on three independent experiments by measuring three plates in each replicate. Asterisk marks a significant difference between the mutants and the P131 strain by using *t*-test (*p* < 0.01). (**G**) Conidium morphology of strains P131 and KSF5−1 stained with Calcofluor white. Bar, 20 μm. (**H**) Statistics on conidium morphology of strains P131, KSF5−1, and KSF5−5. The means were calculated by measuring 100 conidia.

The protein sequence of *S. cerevisiae* Snf5 (YBR289W) was used to search the homologous gene of *M. oryzae* from *Magnaporthe* comparative database with Blast_P. The percentage of sequence identity between *S. cerevisiae* Snf5 and MGG_06315 is 35.85%. A predicted protein MGG_06315 exhibited the highest similarity to ScSnf5 and was named as MoSnf5 in this study, which contains an Snf5 domain in its middle region (Figure S1A). In order to investigate the biological roles of MoSnf5, its gene deletion mutants were generated by introducing a gene knockout vector pKOMoSNF5 into a wild-type strain P131, and two gene deletion mutants, KSF5−1 and KSF5−5, were identified by PCR screening and DNA gel blot analysis (Figure S1B,C). We first evaluated their colony growth on OTA plates. While strain P131 formed a 40 mm colony at 5 d post-inoculation (dpi), the Δ*Mosnf5* ones were 21 mm on average (Figure 1A,B). The Δ*Mosnf5* colonies were also obviously less pigmented (Figure 1A). Moreover, the Δ*Mosnf5* vegetative hyphae had shorter inter-hyphal lengths than the wild-type ones (Figure 1C). When stained with CFW, the average length of the secondary- and tertiary-apical hyphal cells of the Δ*Mosnf5* mutants were 31.6 μm and 34.7 μm, whereas the wild-type ones were 85.7 μm and 92.0 μm, respectively (Figure 1D). Furthermore, we examined whether *MoSNF5* plays a role in cell wall integrity by inoculating the strains onto CM with several chemicals, including 200 μg/mL CFW, 200 μg/mL CR, or 0.05% SDS. While the inhibition rates of strain P131 on CM with CFW and SDS were 23% and 22%, the Δ*Mosnf5* ones were 36% and 37% on average, respectively (Figure S2). These data suggested that *MoSNF5* played important roles in vegetative hyphal growth and cell wall integrity.

We then quantified the capacity on conidiation of the Δ*Mosnf5* mutants. Under a stereomicroscope, seldom conidia were observed on the Δ*Mosnf5* conidiophores cultured on OTA plates, though the Δ*Mosnf5* mutants seemed to produce a similar number of conidiophores as strain P131 (Figure 1E). While strain P131 produced 4 × 10^7^ conidia per plate, the Δ*Mosnf5* mutants had only 6 × 10^5^ conidia under the same condition, which was approximately 1% of the wild-type ones (Figure 1F). Moreover, about 25% of the Δ*Mosnf5* conidia were abnormal in morphology and had only one septum (Figure 1G,H). We also compared expression levels of several conidiation-related transcription regulator genes, including *COS1* [47], *COM1* [36], *CON2* [48], *CON7* [49], *HOX2* [50], and *STUA* [51] between strains P131 and KSF5−1 by RT-qPCR. The results showed that all of these genes were significantly down-regulated in strain KSF5−1 (Figure S3), suggesting that *MoSNF5* positively regulated these conidiation-related genes.

For gene complementation, a genomic fragment containing the open reading frame of *MoSNF5* was introduced into strain KSF5−1. All the 20 complementation transformants, including strain CSF5−12, exhibited the wild-type phenotype (Figure 1), suggesting that the deletion of *MoSNF5* was responsible for the defects of the Δ*Mosnf5* mutants.

### 3.2. MoSNF5 Is Required for Fungal Virulence

**Figure 2:**
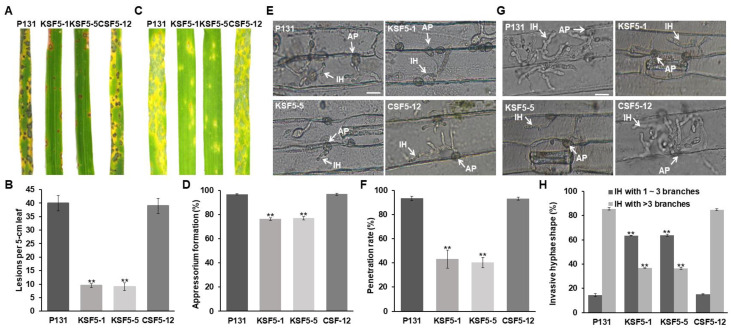
*MoSNF5* is important to fungal virulence. (**A**) Rice seedlings sprayed with conidia of the wild-type strain P131, the Δ*Mosnf5* mutants KSF5−1 and KSF5−5, and its one complementation strain CSF5−12. Typical diseased leaves were photographed at 7 dpi. (**B**) Bar graphs showing the number of disease lesions on 5 cm rice leaf infected by strains P131, KSF5−1, KSF5−5, and CSF5−12. The mean and standard deviations were calculated based on three independent experiments by measuring 10 diseased leaves in each replicate. Asterisk marks a significant difference between the mutants and the P131 strain by using *t*-test (*p* < 0.05). (**C**) Barley seedlings sprayed with conidia of strains P131, KSF5−1, KSF5−5, and CSF5−12. Typical diseased leaves were photographed at 5 dpi. (**D**) Percentages of appressorium formation of strains P131, KSF5−1, KSF5−5, and CSF5−12 on cover glass slides at 12 hpi. The mean and standard deviations were calculated based on three independent experiments by measuring 100 germinated conidia in each replicate. Asterisk marks a significant difference between the mutants and the P131 strain by using *t*-test (*p* < 0.05). (**E**) Appressorial penetration of strains P131, KSF5−1, KSF5−5, and CSF5−12 on barley leaves at 24 hpi. AP, appressoria; IH, invasive hyphae. Bar, 20 μm. (**F**) Percentages of appressorial penetration of strains P131, KSF5−1, KSF5−5, and CSF5−12 on barley leaves at 24 hpi. The mean and standard deviations were calculated based on three independent experiments by measuring 100 appressoria in each replicate. Asterisk marks a significant difference between the mutants and the P131 strain by using *t*-test (*p* < 0.05). (**G**) Invasive hyphae of strains P131, KSF5−1, KSF5−5, and CSF5−12 on barley leaves at 36 hpi. AP, appressoria; IH, invasive hyphae. Bar, 20 μm. (**H**) Percentages of invasive hyphae of strains P131, KSF5−1, KSF5−5, and CSF5−12 on barley leaves at 36 hpi. The invasive hyphae were classified into two main types, 1–3 branches and more than 3 branches. The mean and standard deviations were calculated based on three independent experiments by measuring 100 invasive hyphae in each replicate. Asterisk marks a significant difference between the mutants and the P131 strain by using *t*-test (*p* < 0.05).

To determine the roles of the Δ*Mosnf5* mutants in plant infection, we sprayed conidium suspensions of strains P131, KSF5−1, KSF5−5, and CSF5−12 onto susceptible rice seedlings (Figure 2A). On average, approximately 40 typical disease lesions formed on 5 cm leaves with strains P131 and CSF5−12, whereas about 10 spotted disease lesions were produced on leaves inoculated with the Δ*Mosnf5* conidia (Figure 2B). Similar results were obtained by inoculating the strains onto barley seedlings (Figure 2C). These data suggested that *MoSNF5* played a vital role in fungal virulence. We then investigated defects of the Δ*Mosnf5* mutants on infection-related morphogenesis by inoculating the conidia onto epidermal cells of barley. At 12 hpi, about 80% of the Δ*Mosnf5* conidia formed appressoria, while over 95% of strain P131 did (Figure 2D). At 24 hpi, more than 90% of the wild-type appressoria formed penetration pegs, whereas less than 50% of the Δ*Mosnf5* appressoria penetrated host cells (Figure 2E,F). At 36 hpi, more than 80% of the wild-type invasive hyphae (IH) developed at least four branches. Under the same conditions, over 60% of the Δ*Mosnf5* IH formed no more than three branches (Figure 2G,H). These data suggested that *MoSNF5* played important roles in appressorial penetration and IH development.

### 3.3. Different Regions of MoSnf5 Play Important Roles in Growth, Conidiation, and Virulence

**Figure 3:**
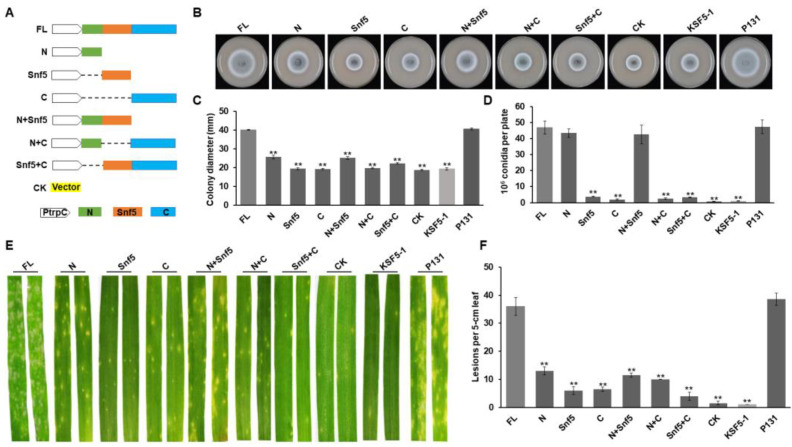
Domain dissection of MoSnf5. (**A**) Strategy on constructing domain deletion vectors of MoSnf5, including the N-terminal region (N), the Snf5 domain (Snf5), the C-terminal region (C), the deletion of the C-terminal region (N+Snf5), the deletion of the Snf5 domain (N+C), and the deletion of the N-terminal region (Snf5+C). The full-length (FL) allele was used as a positive control. The green, orange, and blue boxes represent the N-terminal region, the Snf5 domain, and the C-terminal region of MoSnf5, respectively. All MoSnf5 alleles were driven by the PtrpC promoter and transformed into the Δ*Mosnf5* mutant for phenotypic analysis. (**B**) Colonies of different alleles of MoSnf5 in (**A**) expressed in the Δ*Mosnf5* mutant, the Δ*Mosnf5* mutants KSF5−1, and the wild-type strain P131 cultured on OTA plate at 5 dpi. (**C**) Statistics on colony diameters of strains in (**B**) at 5 dpi. The mean and standard deviations were calculated based on three independent experiments by measuring three plates in each replicate. Asterisk marks a significant difference between the mutants and the P131 strain by using *t*-test (*p* < 0.05). (**D**) Capacity on conidiation of strains in (**B**). The mean and standard deviations were calculated based on three independent experiments by measuring three plates in each replicate. Asterisk marks a significant difference between the mutants and the P131 strain by using *t*-test (*p* < 0.01). (**E**) Barley seedlings sprayed with conidia of strains in (**B**). Typical diseased leaves were photographed at 5 dpi. (**F**) Bar graphs showing number of disease lesions on 5 cm rice leaf infected by strains in (**B**). The mean and standard deviations were calculated based on three independent experiments by measuring 10 diseased leaves in each replicate. Asterisk marks a significant difference between the mutants and the P131 strain by using *t*-test (*p* < 0.05).

MoSnf5 has 771 amino acids with one predicted Snf5 domain in the middle region (181–411 aa) by Pfam prediction (Figure S1A). To figure out the roles of its different regions, including the N-terminal region (1–180 aa), Snf5 domain, and C-terminal region (412–771 aa), we performed region deletion and complementation assays. Six region-deletion complementation vectors were constructed, and the full-length MoSnf5 and empty vector were used as controls (Figure 3A). All constructs were independently introduced into the Δ*Mosnf5* mutants, and at least 10 transformants were obtained for each construct. After analyzing the phenotypes of the resulting transformants, all of them exhibited certain defects on colony growth, conidiation, and plant infection (Figure 3B–F). Among the three regions, the N-terminus exhibited the best effects on rescuing defects of the Δ*Mosnf5* mutants. The constructs harboring the N or the N+Snf5 could almost rescue the capacity on conidiation of the Δ*Mosnf5* mutants (Figure 3D), and showed obviously evaluated capacities on colony growth and plant infection of the Δ*Mosnf5* mutants (Figure 3B,C,E,F). Taken together, these data suggested that different regions of MoSnf5 play important roles in growth, conidiation, and virulence.

### 3.4. MoSnf5 Is a Nuclear Protein and Constitutively Expressed

**Figure 4:**
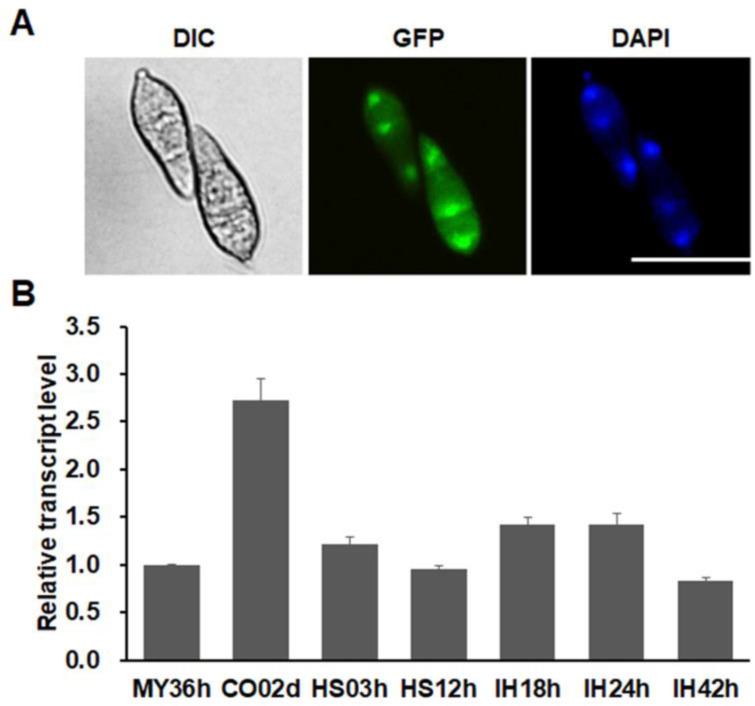
Expression pattern and subcellular localization of MoSnf5. (**A**) Nuclear localization in conidia of a strain expressing the MoSnf5-GFP fusion under the control of a constitutive promoter RP27. DAPI was used to stain the nuclei as a control. Bar, 20 μm. (**B**) The expression levels of *MoSNF5* in vegetative hyphae (HY), conidia (CO), mature appressoria (AP12h), and invasive hyphae (IH48h) were evaluated by q RT-qPCR. Relative abundance of *MoSNF5* transcripts during different developmental stages was normalized by comparing with HY, which was set as 1. Three independent biological replications, each with three technical replicates, were performed.

To determine the subcellular localization of MoSnf5, a *MoSNF5-GFP* fusion driven by its native promoter was generated and introduced into strain KSF5−1. All 15 resulting transformants exhibited wild-type phenotypes, but no fluorescence signals could be observed in these transformants at different developmental stages (data not shown). We then generated a *MoSNF5-GFP* fusion under the control of a constitutively expressed promoter RP27 and introduced it into strain KSF5−1. Similarly, all of the 15 resulting transformants were restored to the wild-type phenotypes, and strong green fluorescence signals were observed in the nucleus of conidia (Figure 4A). These data suggest that MoSnf5 was a nuclear protein. The expression profile of *MoSNF5* was then evaluated by RT-qPCR with RNA samples originating from mycelia, conidia, appressoria, and invasive hyphae. As shown in Figure 4B, *MoSNF5* exhibited relatively low expression levels at all developmental stages, among which the conidia had the highest one (over 2.5-fold than that of mycelia).

### 3.5. MoSNF5 Is Essential to Galactose Utilization and Regulates Expression Levels of Genes of the Leloir Pathway

**Figure 5:**
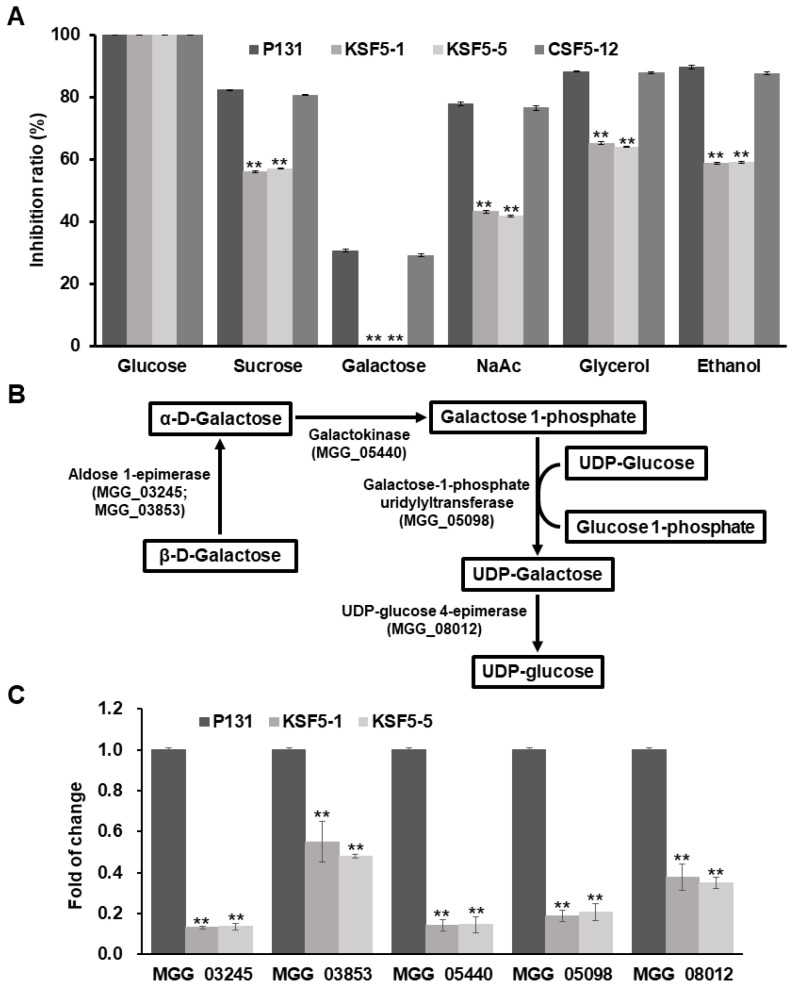
*MoSNF5* is required for galactose utilization and regulates expression levels of genes participating in the galactose metabolism pathway. (**A**) Inhibition ratios of the wild-type strain P131, the Δ*Mosnf5* mutants KSF5−1 and KSF5−5, and its one complementation strain CSF5−12 on minimal agar media supplemented with 0.5 M chemical agents, including glucose, sucrose, galactose, NaAc, glycerol, and ethanol, as the sole carbon source. The mean and standard deviations were calculated based on three independent experiments by measuring three plates in each replicate. Asterisk marks a significant difference between the mutants and the P131 strain by using *t*-test (*p* < 0.05). (**B**) The scheme of the galactose catabolism pathway with four enzymes. Aldose 1-epimerase, MGG_03245 and MGG_03853; galactokinase, MGG_05440; galactose-1-phosphate uridylyltransferase, MGG_05098; UDP-glucose 4-epimerase, MGG_08012. (**C**) Relative expression levels of the six genes participating in the galactose catabolism pathway between strains P131 and the Δ*Mosnf5* mutants KSF5−1 and KSF5−5. The expression level of each gene in strains P131, KSF5−1, and KSF5−5 was normalized against that of the *Actin* gene, and the expression level of each gene in strain P131 was arbitrarily set to 1. The mean and standard deviations were calculated based on two independent experiments. Asterisk marks a significant difference between the mutants and the P131 strain by using *t*-test (*p* < 0.05).

Previously, ScSnf5 was reported to be required for the expression of glucose-repressible genes in *S. cerevisiae*, and the Δ*Scsnf5* mutants had growth defects on non-fermentable carbon sources, such as sucrose, galactose, and glycerol [15]. To test whether MoSnf5 has similar roles in the metabolism of non-fermentable carbon sources, the growth rate of the Δ*Mosnf5* mutants was measured on minimal medium (MM) supplemented with different carbon sources, including glucose, sucrose, galactose, NaAc, glycerol, and ethanol. In comparison with strain P131, the Δ*Mosnf5* mutants exhibited slow colony growth on all tested media except of glucose, which was similar to the Δ*Scsnf5* mutants (Figure 5A). These data suggest that MoSnf5 facilitated similar roles as ScSnf5 in the utilization of carbon sources.

To our surprise, the Δ*Mosnf5* mutants could not grow on a medium with galactose. Due to the Leloir pathway, in which D-galactose is converted to D-glucose-6-phosphate in a redox-neutral way, which was the pathway for galactose catabolism [52], we questioned whether genes of the Leloir pathway (Figure 5B) were regulated by *MoSNF5*. Thus, we compared expression levels of each gene between strains P131 and the Δ*Mosnf5* mutants KSF5−1 and KSF5−5 by RT-qPCR. As expected, all six genes were significantly down-regulated in strains KSF5−1 and KSF5−5 (Figure 5C). So, *MoSNF5* is essential to galactose utilization by positively regulating transcriptional levels of all genes of the Leloir pathway.

### 3.6. MoSnf5 Interacts with Four Proteins via Different Regions

**Figure 6:**
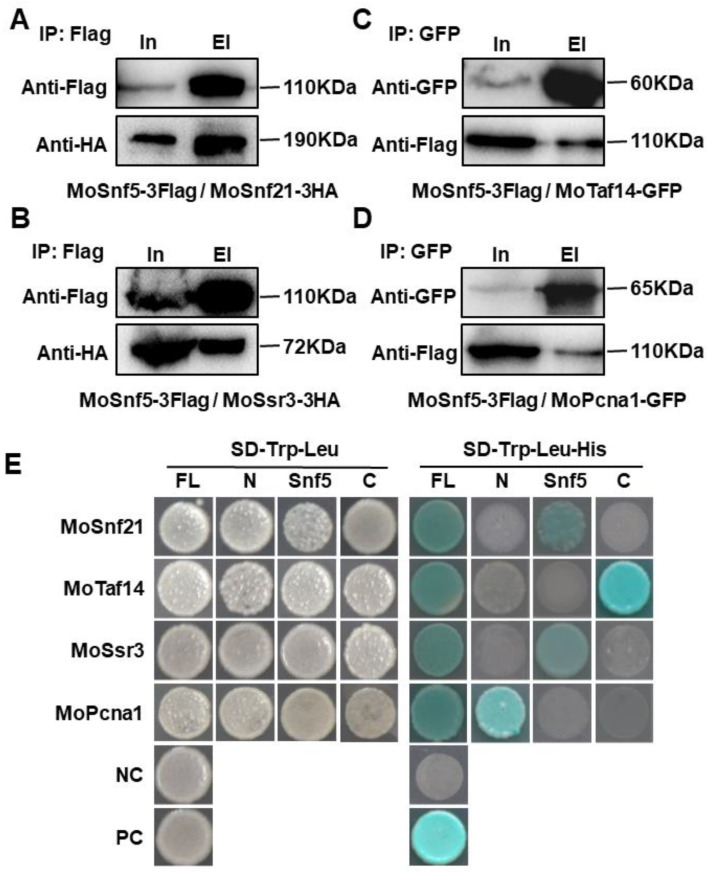
MoSnf5 physically interacts with four proteins via different regions. (**A**,**B**) Co-immunoprecipitation assay on strains expressing MoSnf5-3×Flag and MoSnf21-3×HA (**A**) or MoSsr3-3×HA (**B**). Proteins eluted from anti-FLAG M2 beads were used for Western blot analysis with anti-FLAG or anti-HA antibodies. (**C**,**D**) Co-immunoprecipitation assay on strains expressing MoSnf5-3×FLAG and MoTaf14-GFP (**C**) or MoPcna18-GFP (**D**). Proteins eluted from anti-GFP beads were used for Western blot analysis with an anti-GFP or anti-FLAG antibodies. In, input; EI, elution. (**E**) Physical interaction between MoSnf5 or its different regions and four proteins by yeast two-hybrid approach. The pGBKT7-MoSnf5 or its different regions and pGADT7-MoSnf21, MoTaf14, MoSsr3, or MoPcna1 were co-transformed into yeast strain AH109, and selected on SD-Trp-Leu and SD-Trp-Leu-His plus X-gal. NC, negative control; PC, positive control.

To identify proteins co-purified with MoSnf5, a *MoSNF5*-3×FLAG fusion was generated and transformed into the KSF5−1 strain. Western blot analysis on the resulting transformants displayed an 87 kDa band with an expected size of MoSnf5-3×FLAG protein. By affinity purification, proteins bound to the anti-FLAG M2 beads were eluted and analyzed by LC-MS/MS. About 40 proteins, including several subunits of the SWI/SNF and RSC complex, protein kinases, nucleic acid binding proteins, and cell division control proteins, were repeatedly identified in three independent experiments (Table S3). So, we then selected six proteins with the highest values of the number of significant matches and tested whether they directly interacted with MoSnf5 via a yeast two-hybrid approach (Figure S4). As shown in Figure 6E, four proteins, MoSnf21 (MGG_06388), MoTaf14 (MGG_05204), MoSsr3 (MGG_01794), and MoPcna1 (MGG_00518) were confirmed to directly interact with MoSnf5. Snf21 is a Swi2/Snf2 family ATP-dependent chromatin remodeler and plays an essential role in mitosis in *S. pombe* [53]. Taf14 is a subunit of RNA polymerase II transcription initiation complex D, and is required for the stabilization of the transcription pre-initiation complex [54]. Ssr3 is a subunit of the SWI/SNF and RSC complexes [55]. Pcna1 is a proliferating cell nuclear antigen and functions as a sliding clamp for genomic DNA replication and repair [56,57]. Furthermore, we performed co-immunoprecipitation assay by expressing the MoSnf5-3×FLAG and MoSnf21-3×HA fusion constructs in strain KSF5−1. When proteins eluted from anti-FLAG M2 beads, both an 87 kDa MoSnf5-3×FLAG band and a 166 kDa MoSnf21*-*3×HA band were detected (Figure 6A), suggesting that MoSnf5 was co-immunoprecipitated with MoSnf21. Similarly, MoSnf5 was co-purified with MoSsr3 (Figure 6B). We also performed co-immunoprecipitation assay by expressing the MoSnf5-3×FLAG and MoTaf14-GFP fusion constructs in strain KSF5−1. When proteins eluted from anti-GFP beads, both an 87 kDa MoSnf5-3×FLAG band and a 54 kDa MoTaf14*-*GFP band were detected (Figure 6C), suggesting that MoSnf5 was co-immunoprecipitated with MoTaf14. Similarly, MoSnf5 was co-purified with MoPcna1 (Figure 6D).

To determine the regions of MoSnf5 important for the protein–protein interactions, its N-terminal region, Snf5 domain, and C-terminal region were independently fused with pGADT7 and co-transformed with the pGBKT7-fused MoSnf5-interacting proteins into yeast. As shown in Figure 6E, both MoSnf21 and MoSsr3 interacted with the Snf5 domain, MoPcna1 interacted with the N-terminal region, and MoTaf14 interacted with the C-terminal region. These results suggested that the three regions of MoSnf5 functioned in diverse roles in the interaction with the four interacting proteins.

### 3.7. MoSnf5-Interacting Protein MoTaf14 Is Also Required for Vegetative Hyphal Growth, Conidiation, and Virulence

**Figure 7:**
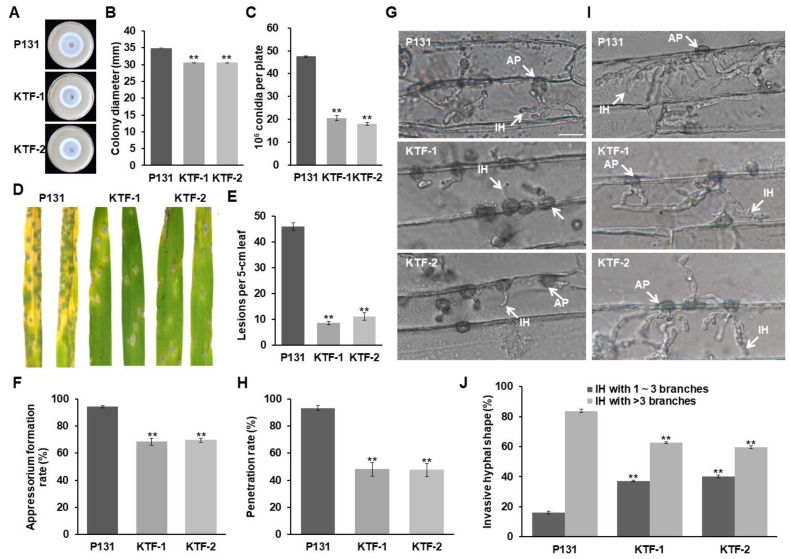
*MoTAF14* is also required for fungal virulence and development. (**A**) Colonies of the wild-type P131, and the *ΔMoTaf14* mutants KTF−1 and KTF−2 cultured on OTA plates at 5 dpi. (**B**) Statistics on colony diameters of strains P131, KTF−1, and KTF−2 at 5 dpi. The mean and standard deviations were calculated based on three independent experiments by measuring three plates in each replicate. Asterisk marks a significant difference between the mutants from strain P131 by using *t*-test (*p* < 0.05). (**C**) Capacity on conidiation of strains P131, KTF−1, and KTF−2. The mean and standard deviations were calculated based on three independent experiments by measuring three plates in each replicate. Asterisk marks a significant difference between the mutants and the P131 strain by using *t*-test (*p* < 0.05). (**D**) Barley seedlings sprayed with conidia of strains P131, KTF−1, and KTF−2. Typical diseased leaves were photographed at 5 dpi. (**E**) Bar graphs showing the number of disease lesions on 5 cm rice leaf infected by strains P131, KTF−1, and KTF−2. The mean and standard deviations were calculated based on three independent experiments by measuring 10 diseased leaves in each replicate. Asterisk marks a significant difference between the mutants and the P131 strain by using *t*-test (*p* < 0.05). (**F**) Percentages of appressorium formation of strains P131, KTF−1, and KTF−2 on cover glass slides at 12 hpi. The mean and standard deviations were calculated based on three independent experiments by measuring 100 germinated conidia in each replicate. Asterisk marks a significant difference between the mutants and the P131 strain by using *t*-test (*p* < 0.05). (**G**) Appressorial penetration of strains P131, KTF−1, and KTF−2 on barley leaves at 24 hpi. AP, appressoria; IH, invasive hyphae. Bar, 20 μm. (**H**) Percentages of appressorial penetration of strains P131, KTF−1, and KTF−2 on barley leaves at 24 hpi. The mean and standard deviations were calculated based on three independent experiments by measuring 100 appressoria in each replicate. Asterisk marks a significant difference between the mutants and the P131 strain by using *t*-test (*p* < 0.05). (**I**) Invasive hyphae of strains P131, KTF−1, and KTF−2 on barley leaves at 36 hpi. AP, appressoria; IH, invasive hyphae. Bar, 20 μm. (**J**) Percentages of invasive hyphae of strains P131, KTF−1, and KTF−2 on barley leaves at 36 hpi. The invasive hyphae were classified into two main types, 1–3 branches and more than 3 branches. The mean and standard deviations were calculated based on three independent experiments by measuring 100 invasive hyphae in each replicate. Asterisk marks a significant difference between the mutants and the P131 strain by using *t*-test (*p* < 0.05).

To investigate the biological roles of MoSnf5-interacting proteins, we selected MoTaf14, which contains a YEATS family and a BET domain (Figure S1D), for further functional analysis. Two knockout mutants of *MoTAF14*, KTF−1 and KTF−2, were generated and confirmed by DNA gel blot (Figure S1E,F). In comparison with strain P131, the Δ*MoTaf14* mutants formed smaller colonies (Figure 7A,B) and produced about a 0.5-fold number of conidia (Figure 7C). Moreover, while strain P131 formed about 43 typical disease lesions per 5 cm barley leaves, the Δ*MoTaf14* mutants just formed about 10 disease lesions (Figure 7D,E). Microscopic observations showed that about 70% of the Δ*MoTaf14* conidia develop appressoria on hydrophobic surfaces at 12 hpi (Figure 7F), and less than 50% of the Δ*MoTaf14* conidia penetrate into host cells at 24 hpi (Figure 7G,H). Moreover, about 40% of the Δ*MoTaf14* appressoria formed IH with no more than three branches at 36 hpi (Figure 7I,J). Taken together, the Δ*MoTaf14* mutants exhibited similar phenotypes as the Δ*Mosnf5* mutants, both of which were deficient in development, infection-related morphogenesis, and plant infection.

### 3.8. Both MoSnf5 and MoTaf14 Are Indispensable for Autophagy

Previous studies showed that core subunits of the SWI/SNF complex in human cells, such as BRG1 and SMARCB1, regulate transcriptional levels of autophagy genes, which are required for the biogenesis of autophagosomes [58,59]. To test whether MoSnf5 and MoTaf14 also play roles in autophagy, we compared the expression levels of 24 autophagy genes in strains KSF5−1 and KTF−1 with those in the P131 strain with RT-qPCR. The results showed that eight autophagy genes were down-regulated and other 16 ones were up-regulated in strain KSF5−1 (Figure S5A), and that seven genes were down-regulated and other 17 ones were up-regulated in strain KTF−1 (Figure S5B). At the level of the whole view, the altered expression levels of autophagy genes in strain KSF5−1 were significantly higher than that in strain KTF−1, and the genes *ATG4*, *ATG5*, *ATG14*, *ATG15*, *ATG16*, and *ATG18* were all up-regulated in both strains KSF5−1 and KTF−1 (Figure S5). These data suggested that both MoSnf5 and MoTaf14 were involved in regulating expression levels of autophagy genes.

**Figure 8:**
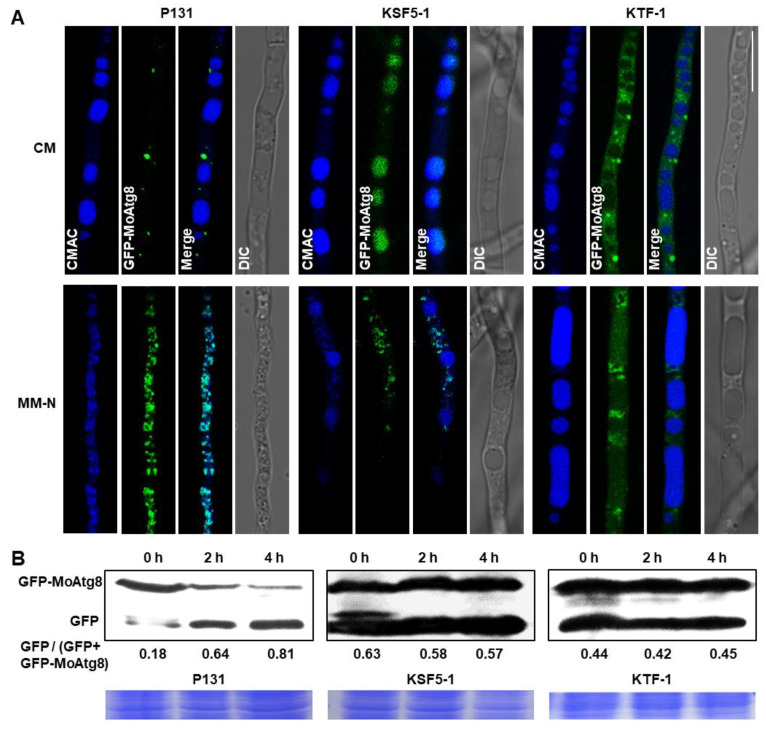
Both MoSnf5 and MoTaf14 are involved in autophagy. (**A**) Subcellular localization of the GFP-MoAtg8 fusion in the wild-type P131, the Δ*Mosnf5* mutant KSF5−1, and the Δ*MoTaf14* mutant KTF−1. The strains were first grown in liquid CM medium for 48 h (upper panel), and then shifted to a nitrogen starvation condition (liquid MM-N medium with 4 mM PMSF) for 4 h (lower panel). Mycelia were stained with CMAC and examined under a fluorescence microscope. Bar, 10 μm. (**B**) The proteolysis of GFP-MoAtg8 in strains P131, KSF5−1, and KTF−1. Mycelia were cultured in liquid CM medium for 48 h (0 h), and then the autophagy was induced under nitrogen starvation conditions for 2 h (2 h) and 4 h (4 h). Mycelia were collected at the indicated time and analyzed by Western blot with an anti-GFP antibody.

In *M. oryzae*, autophagic membrane protein MoAtg8 served as a marker protein of the autophagosome, and the cleavage of GFP-MoAtg8 was widely used to monitor the process of autophagy [46,60,61]. So, we measured subcellular localization of the GFP-MoAtg8 fusion in strains KSF5−1 and KTF−1, and assayed its proteolysis process by treating the strains under a stress condition, MM-N liquid medium with proteinase B inhibitor, phenylmethyl sulfonyl fluoride (PMSF). For strain P131, the GFP-MoAtg8 was dotted in the vicinity of the large CMAC-stained vacuoles under the CM condition, whereas it was co-localized with the punctate vacuoles under the stress condition (Figure 8A). For strain KSF5−1, the majority of the GFP-MoAtg8 was assembled into large structures that overlapped with the vacuoles under the CM condition, whereas the GFP-MoAtg8 was mostly distributed as dots outside of the vacuoles and partially accumulated in vacuoles under the stress condition. For strain KTF−1, the GFP-MoAtg8 was evenly distributed among the cytoplasm and with occasional dot structures under the CM condition; under the stress condition, the GFP-MoAtg8 was partially aggregated as patches outside of the fused vacuoles and partially evenly distributed among the vacuoles (Figure 8A). In proteolysis assays, the GFP-MoAtg8 band was detected with an anti-GFP antibody in strain P131 in CM, and the amount of the GFP-MoAtg8 apparently decreased with time accompanied with increased levels of free GFP under the stress condition (Figure 8B). However, for strains KSF5−1 and KTF−1, the ratio between GFP and the GFP-MoAtg8 showed no obvious changes under normal and stress conditions (Figure 8B). Interestingly, under the CM condition, the ratio of proteolyzed GFP from GFP-MoAtg8 in both strains KSF5−1 and KTF−1 was significantly higher than that in strain P131, which was coincident with the observations on the subcellular localization of the GFP-MoAtg8 (Figure 8). Taken together, these data suggested that both MoSnf5 and MoTaf14 played indispensable roles in the biogenesis of autophagosomes.

## 4. Discussion

In this study, we first reported the biological functions of Snf5 and the underlying mechanisms in plant pathogenic fungi, and found that MoSnf5 plays important roles in fungal growth, conidiogenesis, infection-related morphogenesis, and plant infection in *M. oryzae*. As a core component of the SWI/SNF complex, Snf5 physically interacts with several components of the SWI/SNF complex, including Swi2/Snf2 or Brm (brahma) [7,8]. Similarly, we also find that several components of the SWI/SNF complex are coimmunoprecipitated with MoSnf5, including Swi1, Swi3, Snf21, Ssr3, and Ssr4. Moreover, MoSnf5 physically interacts with one component of the TFIID complex, MoTaf14, through its C-terminus, which is consistent with the previous finding that the SWI/SNF regulators are identified as integral components of the RNA polymerase II holoenzyme in yeast [62]. These findings suggest that the components of the SWI/SNF complex are highly conserved among different eukaryotes. MoSnf1, another core component of the SWI/SNF complex, was also previously reported to facilitate similar biological functions in *M. oryzae* [63]. Thus, we speculate that the SWI/SNF complex is required for the fungal virulence and development in *M. oryzae*, and it deserves to be investigated in the biological roles of other components of the SWI/SNF complex in the future.

For successful infection, *M. oryzae* needs to penetrate host cells by accumulating high glycerol concentration and enormous turgor pressures within the appressorium [29]. Several findings also report that autophagy controls appressorial maturation and penetration, and spatiotemporally-regulated autophagy genes are essential for functional appressorium, including *MoATG1*, *MoATG4*, *MoATG5*, *MoATG8*, and *MoATG9* [64,65]. Our findings showed that about half of the Δ*Mosnf5* appressoria could not efficiently penetrate into host cells, and that the Δ*Mosnf5* mutants exhibited obvious defects in autophagy and significantly altered expression levels of ATG genes. So, we speculate that the deficiency in appressorial penetration of the Δ*Mosnf5* mutants should be mainly caused by impaired autophagy. Recently, the SWI/SNF complex component BRG1 has been found to serve as a key regulator directly governing the transcription of ATG genes, including Atg16l1 and Atg7 [58]. The SWI/SNF complex component SMARCB1 is also identified to function upstream of ATG5-mediated autophagy, and directly binds to the ATG5 promoter and epigenetically inhibits its transcription [59]. In *M. oryzae*, the detailed mechanism of how MoSnf5 regulates the ATG genes and the autophagy remains to be explored.

After successfully penetrating host plant cells with a glucose-rich, nitrogen-poor environment, *M. oryzae* undergoes a profound change in fungal morphology, including thin primary infection hyphae and bulbous secondary invasive hyphae. For survival, *M. oryzae* needs to absorb nitrogen from host cells, including amino acids, purines, and cell wall polysaccharides [33]. Our findings showed that the Δ*Mosnf5* mutants formed significantly less branched invasive hyphae and extended disease lesions. Combined with the findings that the Δ*Mosnf5* mutants could not well utilize different non-fermentable carbon sources in vitro, including galactose and sucrose, we speculate that the Δ*Mosnf5* mutants could not efficiently utilize certain carbon sources (such as galactose and sucrose) from host cells, which eventually leads to defects in invasive hyphal development and attenuated fungal virulence. Interestingly, the transcriptional expression levels of genes participating in galactose catabolism were all down-regulated in the Δ*Mosnf5* mutants, which led to low amounts of 6-phosphate glucose (G6P). Previous findings showed that the availability of G6P by Tps1 is the cardinal process controlling rice blast infection [66]. So, we speculate that the role of MoSnf5 on invasive hyphal development should be partially attributed by Tps1 on the utilization of G6P obtained from host cells.

Our findings show that the Δ*Mosnf5* mutants form smaller colonies and shorter hyphal cells, both of which are about half the size of the wild-type strain. So, we speculate that MoSnf5 is involved in the process of cell polarity but not the cell cycle. It will be interesting to reveal the mechanism underlying how MoSnf5 regulates a specific set of genes related to cell polarity. Moreover, the Δ*Mosnf5* mutants produce approximately 1/50-fold conidia of the wild-type strain, which is caused by the reduced number of conidiophores and the fewer conidia produced on each conidiophore. Therefore, it is interesting to dissect the MoSnf5-regulated genes related to the formation of conidiophores and conidia. Further, the six tested genes related to conidiation are all down-regulated with the deletion of *MoSNF5*. Due to MoSnf5 interacting with the transcription initiation factor TFIID subunit MoTaf14, we speculate that MoSnf5 might regulate the expression levels of the six conidiation-related genes through MoTaf14.

In summary, our findings in this study indicate that *M. oryzae* Snf5 regulates fungal virulence, growth, and conidiation, which are likely through modulating galactose utilization and autophagy.

## Data Availability

The data presented in this study are included in the article; further inquiries can be directed to the corresponding author.

## Supplementary Materials

The following supporting information can be downloaded at: https://www.mdpi.com/article/10.3390/jof9010018/s1, Figure S1: Schematic representation of domains and targeted deletion of *MoSNF5* and *MoTAF14*; Figure S2: *MoSNF5* plays a role in maintaining cell wall integrity; Figure S3: *MoSNF5* regulates expression levels of genes related to conidiation; Figure S4: The MoSnf5-coimmunoprecipitated proteins used for yeast two-hybrid test; Figure S5: Deletion of *MoSNF5* and *MoTAF14* alter the expression levels of autophagy genes; Table S1: Fungal strains used in this study; Table S2: PCR primers used in this study; Table S3: List of proteins coimmunoprecipitated with MoSnf5.
