# Supplementary material for: MoSnf5 Regulates Fungal Virulence, Growth, and Conidiation in Magnaporthe oryzae
Source: J Fungi (Basel). 2022 Dec 22;9(1):18. doi: 10.3390/jof9010018 (PMC9861326; doi:10.3390/jof9010018)
Supplement: Supplementary file 1 [file jof-09-00018-s001.zip › Supplementary Materials.zip!/Supplemental materials legends-1.pdf]

Journal of *Fungi*

MDPI

Supplemental materials

Figure S1. Schematic representation of domains and targeted deletion of *MoSNF5* and *MoTAF14*. (A) Domain scheme of MoSnf5. (B) The strategy of the deletion of *MoSNF5*. The *MoSNF5* gene knockout vector pKNH-SNF5 was constructed by amplifying the upstream and the downstream flanking sequences and ligated with the hygromycin phosphotransferase gene (*hph*) cassette. S, *Sal*I. (C) Southern blot analysis of the *MoSNF5* knockout mutants. The *SalI*-digested genomic DNAs were hybridized with an upstream fragment of *MoSNF5.* The estimated size of each hybridized band was indicated at right. (D) Domain scheme of MoTaf14. (E) The strategy of the deletion of *MoTAF14*. The *MoTAF14* gene knockout vector pKNH-TAF14 was constructed by amplifying the upstream and the downstream flanking sequences and ligated with the *hph* cassette. X, *Xba*I. (F) Southern blot analysis of the *MoTAF14* knockout mutants. The *Xba*I-digested genomic DNAs were hybridized with a downstream fragment of *MoTAF14.* The estimated size of each hybridized band was indicated at right.

Figure S2. *MoSNF5* plays a role in maintaining cell wall integrity. (A) Colonies of the wild-type P131, the Δ*Mosnf5* mutants KSF5-1 and KSF5-5, and its one complementation strain CSF5-12 cultured on CM plate and CM plate supplemented with 200 μg/ml CR, 200 μg/ml CFW, or 0.05%SDS at 5 dpi. (B) The inhibition ratio of colony growth of strains P131, KSF5-1, KSF5-5, and CSF5-12 were cultured on CM plates with 200 μg/ml CR, 200 μg/ml CFW, or 0.05%SDS. The mean and standard deviations were calculated based on three independent experiments by measuring three plates in each replicate. Asterisk marks a significant difference between the mutants and the P131 strain by using *t*-test ($p < 0.05$).

Figure S3. *MoSNF5* regulates expression levels of genes related to conidiation. Relative expression levels of 6 conidiation-related genes in the conidiophores of strains the wild-type strain P131, the Δ*Mosnf5* mutant KSF5-1, and its one complementation strain CSF5-12. The expression level of each gene in strains P131 and KSF5-1 was normalized against that of the *Actin* gene, and the expression level of each gene in strain P131 was arbitrarily set to 1. The mean and standard deviations were calculated based on two independent experiments. Asterisk marks a significant difference between the mutants and the P131 strain by using *t*-test ($p < 0.05$).

Figure S4. The MoSnf5-coimmunoprecipitated proteins used for the yeast two-hybrid test. The MoSnf5-interacting proteins were identified by co-immunoprecipitation and following LC-MS/MS, and then assayed their physical interaction with MoSnf5 via yeast two-hybrid approach.

Figure S5. Deletion of *MoSNF5* and *MoTAF14* alter the expression levels of autophagy genes. (A-B) Expression levels of 24 autophagy genes were down-regulated (A) and up-regulated (B) with the deletion of *MoSNF5*. (C-D) Expression levels of 24 autophagy genes were down-regulated (C) and up-regulated (D) with the deletion of *MoTAF14*. The gene expression levels were evaluated by quantitative real-time RT-PCR. The expression level of each gene in the wild-type strain P131, the Δ*Mosnf5* mutants KSF5-1, and the *ΔMoTaf14* mutants KTF-1 was normalized against that of the *Actin* gene, and the expression level of each gene in strain P131 was arbitrarily set to 1. The mean and standard deviations were calculated based on two independent experiments. Asterisk marks a significant difference between the mutants and the P131 strain by using *t*-test ($p < 0.05$).

Table S1. Fungal strains used in this study.

Table S2. PCR primers used in this study.

Table S3. List of proteins coimmunoprecipitated with MoSnf5.
